# Unusual skin manifestation of hand, foot and mouth disease associated with coxsackievirus A6: cases report

**DOI:** 10.1186/s40064-015-1143-z

**Published:** 2015-07-17

**Authors:** Susheera Chatproedprai, Therdpong Tempark, Nasamon Wanlapakorn, Jiratchaya Puenpa, Siriwan Wananukul, Yong Poovorawan

**Affiliations:** Department of Pediatrics, Faculty of Medicine, Center of Excellence in Clinical Virology, Chulalongkorn University, Bangkok, 10330 Thailand

**Keywords:** Unusual skin presentation, Hand, foot and mouth disease, Coxsackievirus A6

## Abstract

**Background:**

Hand, food, and mouth disease (HFMD) is a highly contagious disease caused by enteroviruses infection. It is a health problem in young children under 5 years of age worldwide. The common causative agents are coxsackievirus A 16 (CA16) and enterovirus 71 (EV71). In recent years, coxsackievirus A6 (CA6) has emerged to be one of the major etiologic agents of HFMD worldwide including in Thailand.

**Case description:**

We reported cases with unusual skin manifestations of CA6-associated HFMD such as widespread severe cutaneous eruption, large vesicles (varicelliform), purpuric-like lesions or Gianotti–Crosti like eruptions.

**Discussion and evaluation:**

Molecular characterization of the CA6 strains from those patients found that all were clustered in the same group of CA6 that are currently circulating in Thailand.

**Conclusions:**

Clinicians need to be aware of the expanded range of cutaneous findings in CA6-associated HFMD in order to properly consider the diagnosis, management and prevention.

## Background

Hand, food, and mouth disease (HFMD) is a highly contagious disease caused by enteroviruses infection. It is a health problem in young children under 5 years of age worldwide. The common causative agents are coxsackievirus A 16 (CA16) and enterovirus 71 (EV71). In recent years, coxsackievirus A6 (CA6) has emerged to be one of the major etiologic agent of HFMD worldwide including a large scale outbreak in Thailand (Puenpa et al. 2013). The typical clinical manifestations of HFMD are fever, multiple ulcers in the throat and soft palate, accompanied by rash or small vesicles on palms and soles. Our objective is to present atypical cutaneous manifestations of HFMD caused by CA6. Those findings will be useful for pediatricians in order to diagnose and make a differential diagnosis of HFMD.

## Case presentation

The cases exemplified atypical HFMD caused by the emerging CA6. Symptoms were characterized by fever, mouth lesions and unusual skin manifestation (Table 1). All except two individuals were out-patient cases presented to our hospital with unusual presentation of HFMD, which were included for further laboratory investigation for enterovirus molecular diagnosis during June–September 2014. All were previously well except for case 7 (who had underlying acute lymphoblastic leukemia; ALL) and all had no previous eczema or other skin conditions. The study was approved by the IRB of the Faculty of Medicine, Chulalongkorn University and adheres to the provisions outlined in the Declaration of Helsinki (IRB 1169/2557).

**Table 1:** Summary of clinical presentation and laboratory investigation of coxsackievirus A6-associated HFMD cases

Case	Sex	Age (years)	History of contact	Clinical manifestation				Laboratory investigation				Remarks
				Oral	Hands/feet	Buttock	Unusual presentation	Tzanck smear	CA6-PCR			
									Throat swab	Stool	Vesicles/lesions	
1	F	10 months	+	+	+	+	WAL, large vesicles	NA	+	NA	NA	
2	M	1	−	+	+	+	WAL, large vesicles	NA	+	+	+	Desquamation
3	M	11 months	−	+	+	+	WAL, large vesicles	−	+	NA	+	Desquamation
4	F	10	−	−	+	−	Crust on scalp	NA	+	NA	+ (crust on scalp)	
5	M	12	−	+	+	+	WAL, purpuric-like, crust on scalp	NA	+	NA	NA	Desquamation
6	F	2	+	−	+	+	GCS-like	NA	+	NA	NA	
7	F	10	−	−	+	−	WTB, face (perioral), purpuric-like	−	+	+	NA	Underlying ALL, desquamation
8	M	10 months	−	+	+	−	GCS-like, face (perioral)	−	−	+	NA	

*CA-6* coxsackievirus A6, *F* female, *M* male, *NA* not available, *WAL* widespread (arms, legs), *GCS* Gianotti–Crosti syndrome, *WTB* widespread (trunk, back).

### Enterovirus diagnosis

**Figure 1:**
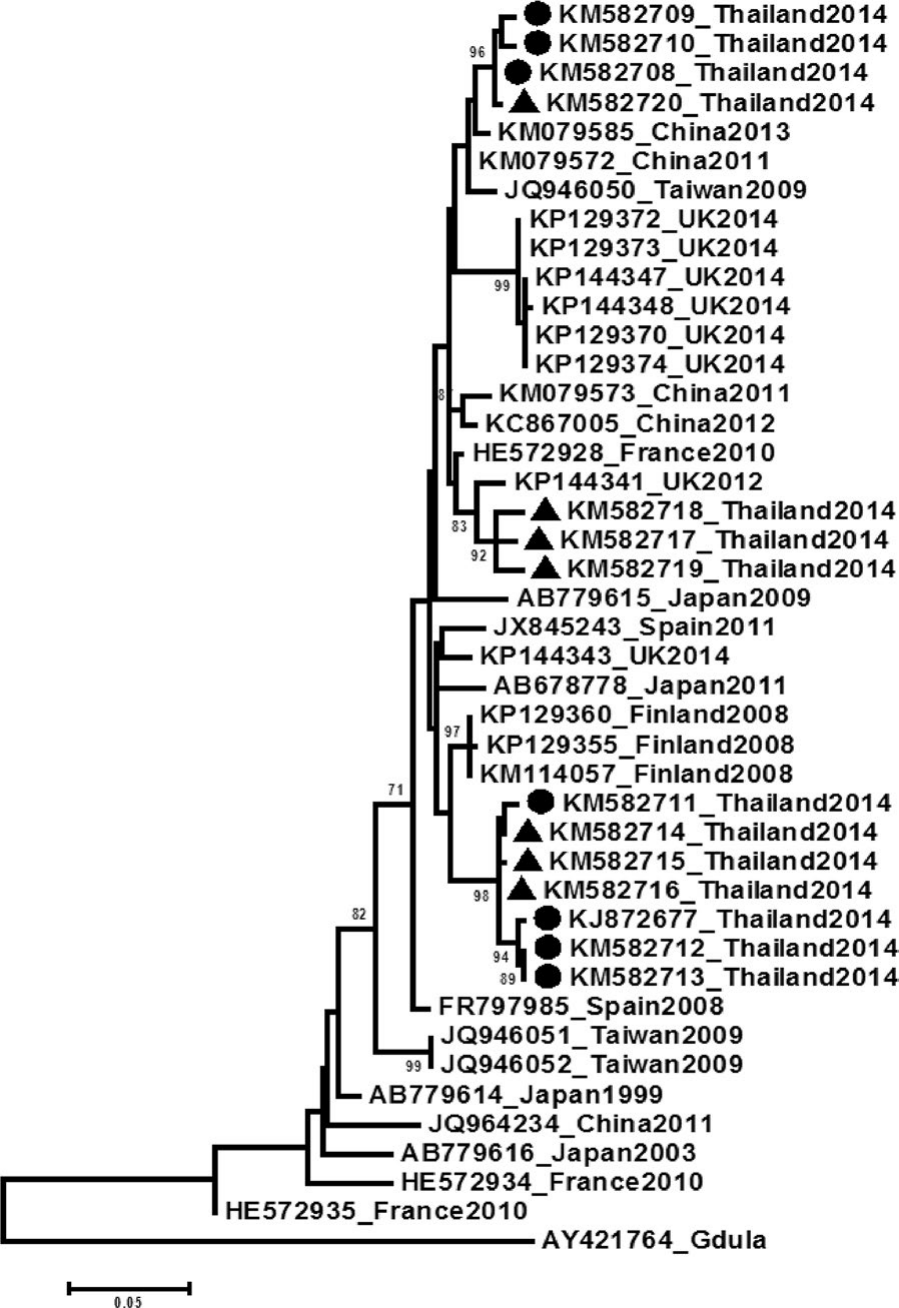
Phylogenetic relationship for coxsackievirus A6 strains detected in this study (viral protein 1 region, positions 2,630–3,051). The tree was constructed through the maximum likelihood method using the best model, Kimura’s two parameter (K2P) + gamma distribution (G). Gdula strain AY421764 was used as reference. Bootstrap re-sampling was used to determine robustness of groupings; values of ≥70% shown. *Scale bar* indicate the number of substitutions per nucleotide position. The strains in this study are indicated in *circle* and contemporary strains detected in Thailand in cases of typical HFMD in *triangle*.

Clinical specimens (throat swab and/or vesicular fluid and/or stool) were processed and subjected to molecular diagnosis as described elsewhere (Puenpa et al. 2013). Samples of all patients were positive using two reverse transcription-polymerase chain reaction (RT-PCRs) methods: one for detecting the 5´ unstranslated region of enterovirus (pan-EV) and the other to detect the viral protein 1 (VP1) region of coxsackievirus A6. Genotyping was done by direct sequencing of the product of the second PCR (420 nt). Nucleotide sequences of the VP1 region were imported and aligned using the ClustalW program. A phylogenetic tree of the partial VP1 region was constructed in the Mega version 6 software through the Maximum Likelihood method using the best model (Tamura et al. 2013). The nucleotide sequences obtained were deposited in the GenBank databases under the accession numbers KJ872677 and KM582708-KM582713. Sequences of seven contemporary CA6 strains detected in Thailand in cases of typical childhood HFMD were included for the phylogenetic analysis (KM582714-KM582720). The Gdula reference strain (GenBank accession number AY421784) and other representative strains were also included in the analysis. All CA6 strains causing atypical HFMD identified in this study grouped into two groups with contemporary strains detected in Thailand (Figure 1).

### Case 1

**Figure 2:**
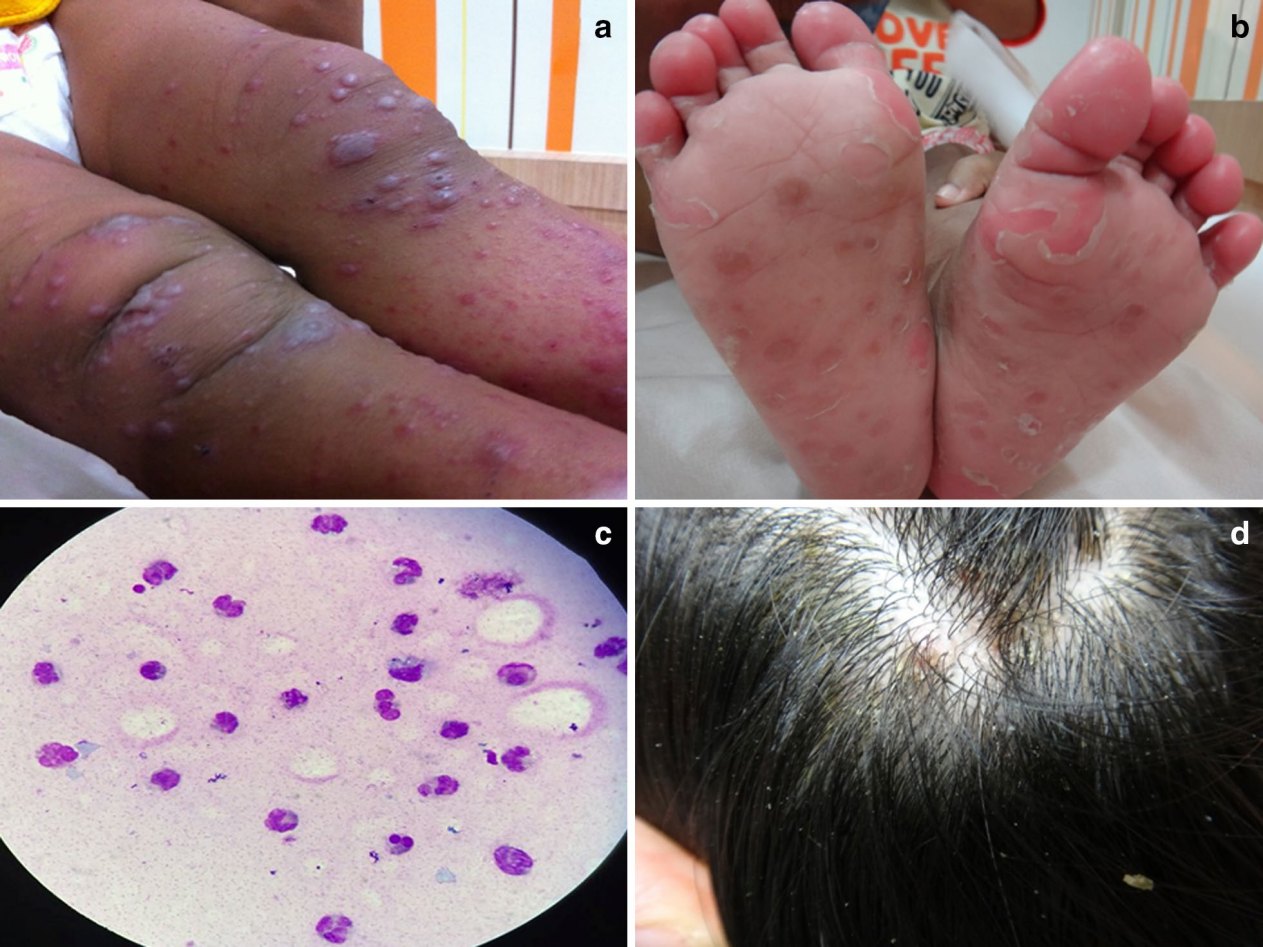
Dermatologic findings of unusual HFMD **a** varicelliform lesions, **b** delayed desquamation, **c** Tzanck smear, **d** on scalp.

A 10-month-old girl presented with fever, cough and excessive drooling for a day. She was otherwise healthy and had completed her scheduled immunization for her age. She had been in close-contact with two toddlers (ages 2 and 3 years old) with active HFMD from her neighborhood 3 days prior to the onset of symptoms. Physical examination on admission revealed slight fever (38.3°C), multiple small ulcers on the soft palate and multiple vesicles on erythematous base on the palms and soles. She was provided with supportive care (acetaminophen syrup to reduce the discomfort). A day later, multiple small (<5 mm) and large vesicles (5–9 mm) rapidly developed on the arms, legs and buttocks. The large vesicles resembled varicella (varicelliform) eruption (Figure 2a). No skin lesions were observed on face, trunk and back.

### Case 2

A 1-year-old boy presented with low-grade fever and rash on hands and feet. Physical examination revealed injected pharynx, shallow ulcers in buccal cavity and multiple vesicles on palms and soles. He was treated with acetaminophen syrup. His cutaneous lesions progressively developed to generalized small and large vesicles involving arms, legs, knees, elbows, buttock and purpura-like eruption on palms and soles. Two weeks after the vesicular eruption, he experienced desquamation on his soles (Figure 2b).

### Case 3

An 11-month-old boy presented with rash on body without fever. Physical examination demonstrated widespread vesicular eruption on arms, legs, hands, feet, buttock and ears. He also had large vesicles on arms, legs and buttock. Mouth ulcers were also detected. His mother worried about varicella infection. Tzanck smear was done and showed multiple neutrophils and mononuclear cells without multinucleated giant cell (Figure 2c). He had delayed desquamation on his soles after rash disappeared.

### Case 4

A 10-year-old girl presented with low grade fever, vesicles on her palms and soles for 3 days. No oral lesions were detected. A day later, she developed multiple lesions on her scalp. Physical examination revealed oval-shaped vesicles on an erythematous base on the palms and feet. Generalized multiple discrete erythematous papules with yellowish crust were observed on the scalp (Figure 2d). No skin lesions were observed on face, trunk and buttock. Throat swab specimen and swab from scalp lesions were collected.

### Case 5

**Figure 3:**
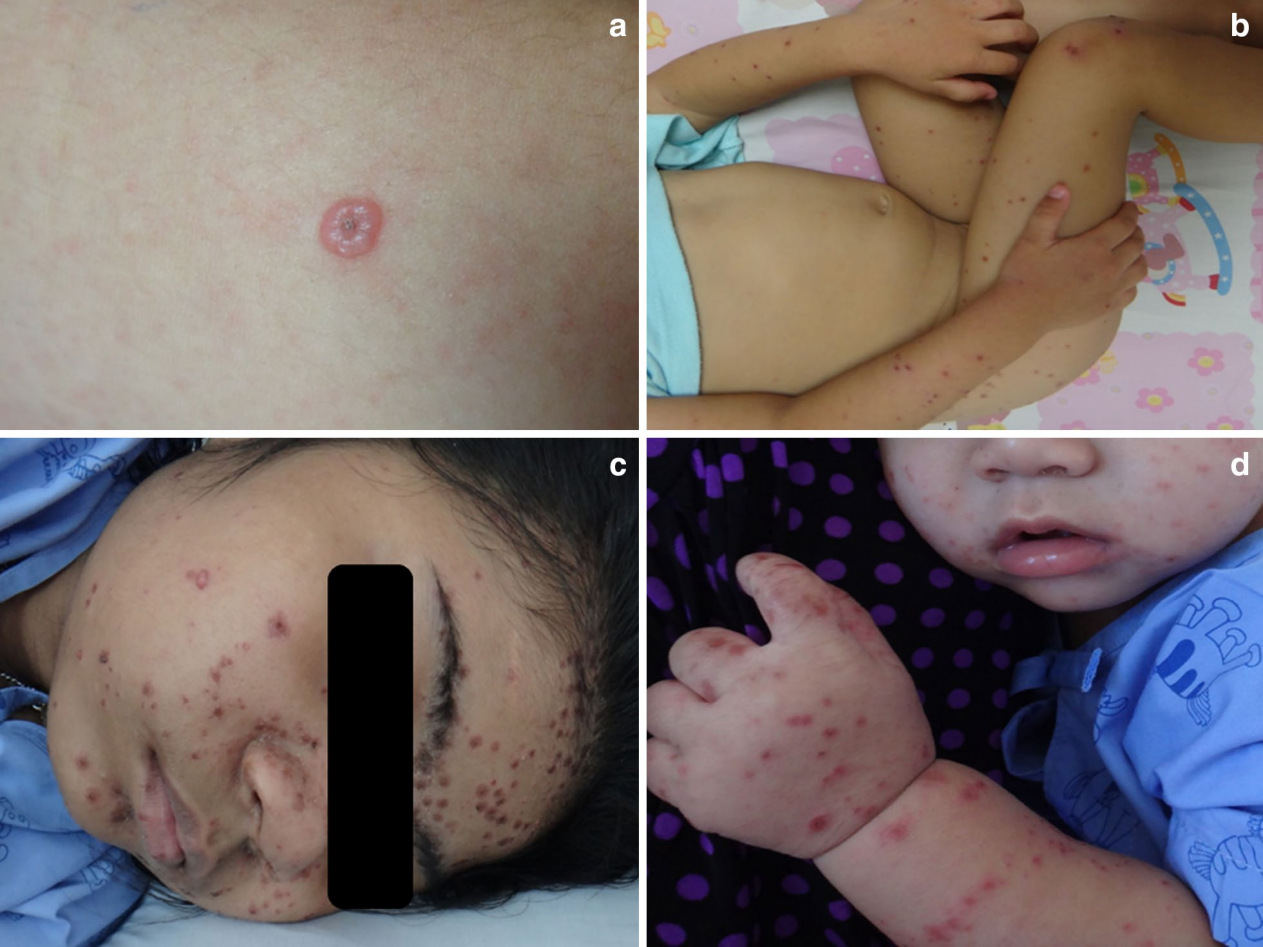
Dermatologic findings of unusual HFMD **a** umbilicated vesicle, **b** Gianotti–Crosti-like, **c** on face, **d** perioral.

A 12-year-old boy presented with low-grade fever, painful mouth ulcer and rash for 2 days. Physical examination showed two shallow ulcers on floor of the mouth. Generalized multiple erythematous papules and purpura-like lesions were present on the trunk and the palms and soles. Vesicles and large umbilicated vesicles were developed on both arms (Figure 3a) and buttock. Few yellowish crusts on an erythematous base were detected on his scalp.

### Case 6

A 2-year-old girl presented with rash on buttock and extremities for 4 days. Firstly the rash began as multiple vesicles on buttock and extremities and then changed into erythematous papules and crust on top. She has been in contact with two friends who had the same rash at school. She was diagnosed as chickenpox infection from a doctor. Physical examination demonstrated dry erythematous papules on arms, legs and buttock with some desquamation on buttock. Few red macules were present on soles. No buccal and trunk lesions were detected (Figure 3b). The cutaneous lesions in this case were presented as Gianotti–Crosti syndrome (GCS)-like because GCS is characterized by monomorphous lichenoid papules and/or papulovesicles, symmetrically distributed on the face, arms and legs with relative sparing of the trunk. However, the lesions in this case disappeared in a week.

### Case 7

A 10-year-old girl with underlying high risk ALL on chemotherapy in maintenance phase presented with itchy vesicles on face without fever and associated symptoms. She was diagnosed as varicella infection and treated with oral acyclovir at the local hospital. The lesions progressed to trunk and back. She was transferred to our hospital. Physical examination on admission demonstrated few vesicles interspersed with erythematous papules and crust on top predominantly on face (Figure 3c), some lesions on trunk, back and extremities. Purpuric-like lesions was found on both palms and soles. Laboratory investigation showed absolute neutrophil count 2,200/mm^3^, Tzanck smear of vesicles did not show multinucleated giant cell. In the meantime of PCR result, high dose intravenous acyclovir (1,500 mg/m^2^/day) was administered to treat as varicella infection. Acyclovir was discontinued after the RT-PCR result. The lesions began to dry and desquamate.

### Case 8

A 10-month-old boy presented with fever, cough and rash on his palms. Physical examination revealed a febrile boy with injected pharynx and shallow ulcers at posterior pharynx. Erythematous macules were found on palms and soles. There was no lesion on the trunk. He was suspected of HFMD with differential diagnosis of GCS. While waiting the diagnostics test results, erythematous macules changed into vesicles and progressively involved face (perioral, Figure 3d), arms and legs.

## Discussion

Hand, foot, and mouth disease is commonly caused by CA16 and EV71 with typical lesions on the hands, feet, mouth, buttock, elbows and knees (Chatproedprai et al. 2010; Hubiche et al. 2014). CA6 emerged as a large scale outbreak in Thailand in 2012 and continued to be one of the major causes of HFMD worldwide (Wei et al. 2011; Flett et al. 2012; Fujimoto et al. 2012; Chung et al. 2013; Kaminska et al. 2013; Lott et al. 2013; Ben-Chetrit et al. 2014; Feder et al. 2014; Hubiche et al. 2014; Li et al. 2014; Sinclair et al. 2014). Unusual manifestations include widespread vesiculobullous and erosive lesions extending beyond the palms and soles (Wei et al. 2011; Chung et al. 2013; Kobayashi et al. 2013; Lott et al. 2013; Mathes et al. 2013; Feder et al. 2014; Hubiche et al. 2014) including dorsal sides of the hands and feet (Flett et al. 2012; Feder et al. 2014), calves and trunk (Feder et al. 2014), eczema herpeticum-like eruption termed “eczema coxsackium” (Mathes et al. 2013; Akkoyunlu et al. 2014; Sinclair et al. 2014), eruption similar to GCS (Mathes et al. 2013), petechiae or purpuric eruption (Mathes et al. 2013; Stewart et al. 2013), varicelliform (Yasui et al. 2013; Sinclair et al. 2014), delayed desquamation of palms and soles (Wei et al. 2011; Mathes et al. 2013), and onychomadesis (transient separation of proximal nail plate) during convalescence (Wei et al. 2011; Feder et al. 2014).

In the present study, all seven of CA6 variants associated with atypical HFMD in Thailand in 2014 were investigated by phylogenetic relationship analysis, which was performed by comparing nucleotide sequences of the VP1 region with previously published sequences. Our results showed that all Thai CA6 variants were closely related to variants associated with previous HFMD outbreaks in Finland, Taiwan and UK (Lo et al. 2011; Österback et al. 2009; Sinclair et al. 2014).

We reported CA6-associated HFMD cases with unusual presentation. Firstly we reported widespread vesicular eruptions (case 1, 2, 3, 5, 7) which can be defined as more than 5 sites involvement (Hubiche et al. 2014) or more than 5% body surface area involvement (Mathes et al. 2013). Furthermore, 2 patients in our series (case 4, 5) had lesions on the scalp which presented as yellowish crust. We confirmed the diagnosis in case 4 by collecting specimens from both throat swab and scalp lesions which all showed CA6. According to widespread vesicular lesions, many authors reported the relationship to CA6 including our cases (Kobayashi et al. 2013; Lott et al. 2013; Mathes et al. 2013; Yasui et al. 2013; Feder et al. 2014; Hubiche et al. 2014). We also reported perioral rash in an immunocompromised patient (case 7) and a healthy 10-month-old boy (case 8).

Dermatological manifestation of HFMD can vary from the classic erythematous papules, vesicles, erosions (usually oval shaped and small) to atypical manifestations including widespread distribution, varicelliform and group of vesicles (Mathes et al. 2013). Lesions are commonly found on the hand, feet and the buccal cavity. Other areas where lesions can be found include buttocks, elbows and knees (Chatproedprai et al. 2010; Hubiche et al. 2014). These clinical features can be used to differentiate HFMD from other common viral infection such as varicella, eczema herpeticum and GCS. Although some patients in our series (case 1, 2, 3) had varicelliform lesions, diagnosis of varicella was unlikely because the distribution of lesions was concentrated on the distal part (limbs and buttocks) rather than on the central area (trunk and back) as in varicella infection. Previous study has described that in both varicella and HFMD, the lesion can be a vesicle followed later by a crust (Hubiche et al. 2014). Differentiation between varicella and HFMD depend on the clinical presentation of the different stages in which vesicles and crusts are found. Only in one-third of HFMD do they occur (Hubiche et al. 2014). However, in the case in which the diagnosis is still in doubt, further laboratory validations such as Tzanck smear (multinucleated giant cells), viral culture, and polymerase chain reaction (PCR) can be helpful.

We also reported a case with underlying ALL (case 7) whose cutaneous lesions were confined to face, trunk and extremities without oral ulcers. Varicella infection was suspected even though we did not notice various stages of lesions as in typical varicella infection in normal host. Treatment with acyclovir was started while waiting for further investigations.

We described 2 cases with purpuric-like lesions (case 5, 7) which were more common in children older than 5 years (Mathes et al. 2013). In addition, we reported 2 cases presenting with GCS-like lesions (case 6, 8). Classic GCS is characterized by monomorphous lichenoid papules and/or papulovesicles, symmetrically distributed on the face, arms and legs with relative sparing of the trunk. Epstein–Barr virus and hepatitis B virus are the most commonly reported causes of GCS (Brandt et al. 2006), but enteroviruses such as coxsackieviruses A16, B4, and B5 have also been implicated (James et al. 1982). The differentiation from HFMD depends on clinical features in that GCS usually spares the palms, soles as well as the mucosa. While the duration of HFMD is usually a week, GCS lasts significantly longer (2–8 weeks).

All of our patients did not have any serious consequences. Only 2 cases were admitted due to underlying disease (case 7) and dehydration (case 8). Four out of eight patients experienced delayed desquamation of palms and soles which was reported in Mathes EF’s (Mathes et al. 2013) and Wei SH’s (Wei et al. 2011) studies.

In our experience, cutaneous manifestations in CA6 HFMD cases were significantly more severe in terms of body surface area involvement and size of lesions than in CA16 and EV71 HFMD cases (Chatproedprai et al. 2010; Puenpa et al. 2011). However, Hubiche et al. (2014) reported that it was difficult to distinguish between infections caused by CA6 and CA16 based on clinical assessments alone, except for perioral rash which was significantly more common in CA6 infection. In EV71 HFMD cases we could not differentiate cutaneous findings from other causative viruses but EV71 cases had higher potential of severe complication such as neurological complication and myocarditis (Chatproedprai et al. 2010).

## Conclusions

CA6-associated HFMD can present with unusual cutaneous manifestations. Clinicians need to be aware of the expanded range of cutaneous findings in CA6-associated HFMD in order to properly consider the diagnosis. Establishing differential diagnoses is necessary in order to prevent misdiagnosis and inappropriate treatment. Hence, it is of great importance to develop rapid and reliable diagnostic methods in order to differentiate and identify the associated viruses with HFMD from other viruses (Kaminska et al. 2013).

## Consent

Written informed consent was obtained from the guardians of patients for the publication of this report and any accompany images.
